# Production of S-methyl-methionine using engineered *Saccharomyces cerevisiae* sake K6

**DOI:** 10.1093/jimb/kuad026

**Published:** 2023-08-31

**Authors:** Jun-Min Lee, Min-Ho Park, Bu-Soo Park, Min-Kyu Oh

**Affiliations:** Department of Chemical & Biological Engineering, Korea University, Seoul 136-763, Korea; Department of Chemical & Biological Engineering, Korea University, Seoul 136-763, Korea; Department of Chemical & Biological Engineering, Korea University, Seoul 136-763, Korea; Samyang Corp. 295 Pangyo-ro, Bundang-gu, Seongnam-si, Gyeonggi-do 13488, Republic of Korea; Department of Chemical & Biological Engineering, Korea University, Seoul 136-763, Korea

**Keywords:** S-methyl-methionine, Methioinine S-methyltransferase, S-adenosyl-methionine, *Saccharomyces cerevisiae* sake K6

## Abstract

S-methyl-methionine (SMM), also known as vitamin U, is an important food supplement produced by various plants. In this study, we attempted to produce it in an engineered microorganism, *Saccharomyces cerevisiae*, by introducing an *MMT* gene encoding a methionine S-methyltransferase from *Arabidopsis thaliana*. The *S. cerevisiae* sake K6 strain, which is a Generally Recognized as Safe (GRAS) strain, was chosen as the host because it produces a significant amount of S-adenosylmethionine (SAM), a precursor of SMM. To increase SMM production in the host, *MHT1* and *SAM4* genes encoding homocysteine S-methyltransferase were knocked out to prevent SMM degradation. Additionally, *MMP1*, which encodes S-methyl-methionine permease, was deleted to prevent SMM from being imported into the cell. Finally, *ACS2* gene encoding acetyl-CoA synthase was overexpressed, and *MLS1* gene encoding malate synthase was deleted to increase SAM availability. Using the engineered strain, 1.92 g/L of SMM was produced by fed-batch fermentation.

**One-Sentence Summary:**

Introducing a plant-derived *MMT* gene encoding methionine S-methyltransferase into engineered *Saccharomyces cerevisiae* sake K6 allowed microbial production of S-methyl-methionine (SMM).

## Graphical Abstract

**Graphical Abstract:**
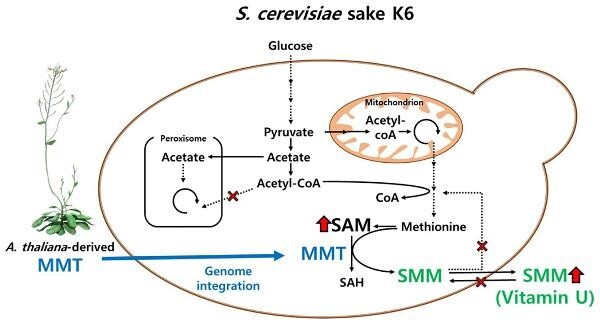
Overall scheme of S-methyl-methionine (SMM) biosynthesis pathway for engineered *Saccharomyces cerevisiae* sake K6.

**Abbreviations**
MMT: Methionine S-methyltransferase
SAM: S-adenosyl-methionine
SMM: S-methyl-methionine
SAH: S-adenosyl-homocysteine

## Introduction

S-methyl-methionine (SMM) is commonly found in plants such as cabbage, broccoli, wheat, and sunflowers (Hattula & Granroth, 1974; Skodak et al., 1965; Turner & Shapiro, 1961). Although its biological role in plants is not well understood, SMM is known to preserve methionine, serve as a methyl donor, and regulate S-adenosylmethionine (SAM) (Kocsis et al., 2003; Tan et al., 2010). In humans, SMM is sometimes referred to as vitamin U because of its therapeutic effects on gastrointestinal ulcers (Cheney, 1949; Samson, 1971; Kopinski et al.,^ 2007^; Sokmen et al., 2012; Gezginci-Oktayoglu et al., 2016; Kruchinina et al., 2018; Topaloglu et al., 2022). Recent studies have shown that SMM is effective in protecting against ultraviolet (UV) exposure and that SMM treatment promotes the growth of human fibroblasts and migration of human dermal fibroblasts, which are essential steps in treating skin wounds (Kim et al., 2018, 2015, 2010). Given these benefits, the demand for SMM as a food supplement has continuously increased.

SMM is commonly produced in flowering plants up to 0.3–5 μmol/g in various organs, such as leaves, roots, etc. (Kovatscheva & Popova,^ 1977^; Bourgis et al., 1999; Kim, 2003; Ludmerszki et al., 2014). It is mainly produced in leaves by methionine S-methyltransferase (MMT) and is transported to reproductive tissues via the phloem. SMM is converted back to methionine through the SMM cycle by homocysteine S-methyltransferase (HMT). This process is called the SMM cycle, and is used for the efficient transport and utilization of methionine during seed development (Cohen et al., 2017; Menegus et al., 2004; Ranocha et al., 2001). Similar to other vitamins, SMM is produced by high-temperature extraction from plants or enzyme synthesis. To date, no studies have investigated conducted on SMM production from microorganisms. In the present study, we introduced *MMT* gene, which encodes a methionine S-methyltransferase from *Arabidopsis thaliana*, into *Saccharomyces cerevisiae* to produce SMM.

**Fig. 1.**
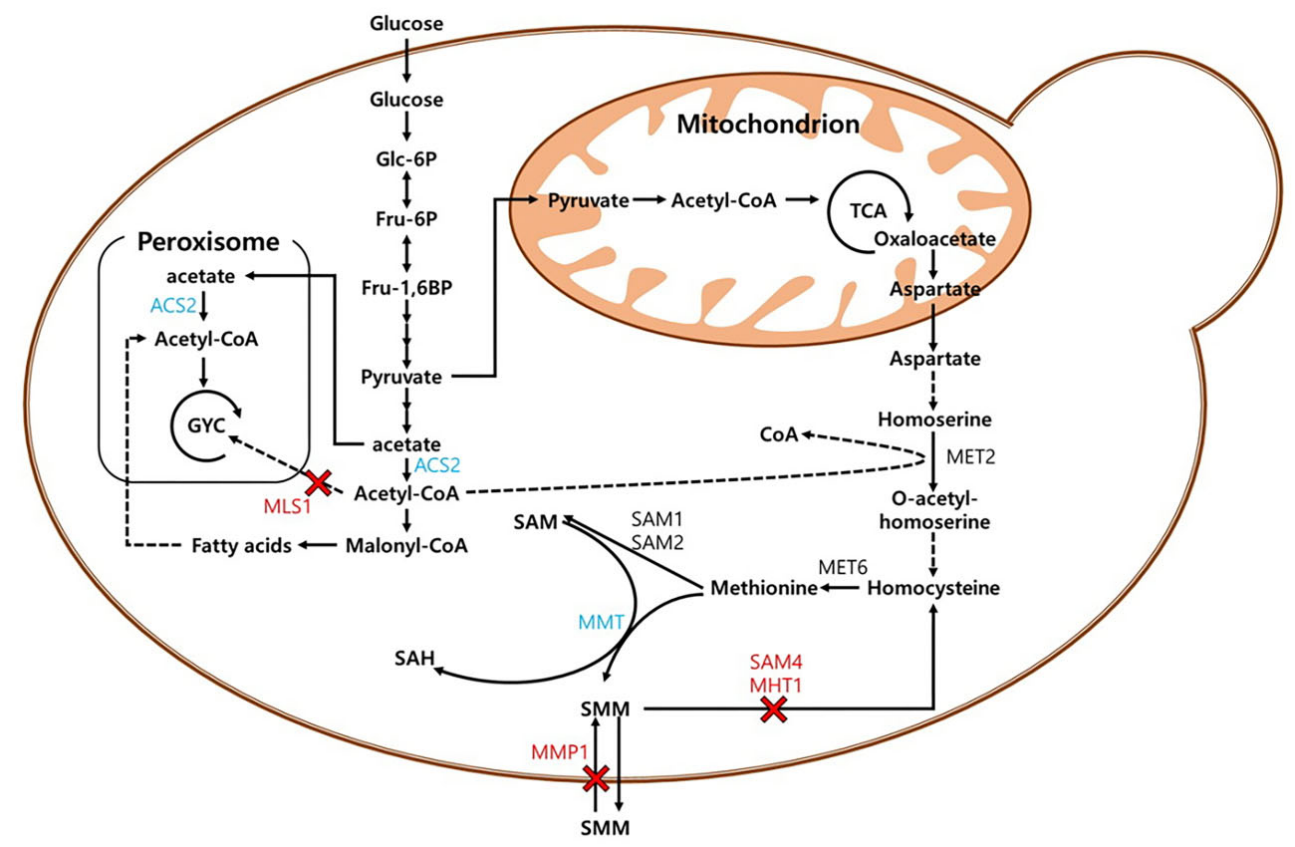
SMM biosynthesis pathway in *S. cerevisiae* sake K6. *MMT* gene encoding methionine S-methyltransferase was integrated to genome. Three genes (*MMP1, SAM4*, and *MHT1*) were knocked out for accumulation of SMM. The native *ACS2* promoter was changed to *PGK1* promoter and *MLS1* gene was knocked out to enhance SAM concentration. The blue colors are up-regulated genes and red colors are knocked out genes. Glc-6p, Glucose-6-phosphate; Fru-6p, Fructose-6-phosphate; Fru-1,6BP, Fructose-1,6-bisphosphate; SAM, S-adenosyl-methionine; SAH, S-adenosyl-homocysteine; SMM, S-methyl-methionine.

*Saccharomyces cerevisiae* has a long history of use in various industries, such as wine making, baking, and brewing. It is a well-studied model organism in eukaryotic biology and the most commonly used microorganism in fermentation processes. The SMM production pathway involves the transfer of a methyl group from SAM to methionine via methionine S-methyltransferase (Fig. 1). The intracellular concentrations of the methionine and SAM precursors play an important role in SMM production. Sake yeast accumulates SAM intracellularly at higher concentrations than other commonly used *S. cerevisiae* strains (Shiozaki et al., 1984). Although there are differences in nutrient characteristics between *S. cerevisiae* sake strains and laboratory strains like S288C, their open reading frames share a high degree of similarity, with an average BLAST similarity of over 95% (Akao et al., 2011). Therefore, we selected the *S. cerevisiae* sake K6 strain for metabolic engineering to produce SMM. In a previous study, an auxotrophic *S. cerevisiae* sake K6 was constructed, making it a host strain for metabolic engineering (Choi et al., 2009). In this study, a *MMT* gene was introduced into the strain to enable the production of SMM.

Several steps were performed to increase SMM production and prevent its degradation by microorganisms. First, we deleted the *MHT1* and *SAM4* genes encoding homocysteine S-methyltransferase to prevent the decomposition of SMM from being decomposed. Additionally, we deleted *MMP1*, which encodes SMM permease, to prevent SMM from being imported into the cells. To further increase the pool of SAM, we upregulated *ACS2*, which encodes acetyl-CoA synthase, by changing the promoter and deleting *MLS1*, which encodes malate synthase (Chen et al., 2016, 2021; Nielsen, 2014). We cultivated this engineered strain to improve SMM production and optimize culture conditions for fed-batch fermentation. Finally, for the first time, high levels of SMM production were achieved using an engineered microorganism.

## Materials and Methods

### Strains and Plasmids

Table 1 provides a list of the strains and plasmids utilized in this study. The *S. cerevisiae* sake K6 (Sake Kyokai No. 6) was purchased from korean collection for type cultures (Daejeon, South Korea). We previously developed a leucine auxotroph for *S. cerevisiae* sake, K6-1 (Choi et al., 2009). *Escherichia coli* DH5α was used for plasmid construction. To construct p425-*MMT*, a codon-optimized *MMT* gene from *A. thaliana* was introduced into the p425GPD plasmid. The amplification of the *MMT* gene and vector backbone fragments was performed using Q5 DNA polymerase (New England Biolabs, MA, USA). The plasmid backbone was assembled by utilizing Gibson Assembly Master Mix (New England Biolabs, MA, USA). To construct the plasmid for CRISPR-Cas9, the pML104-Leu plasmid was used as the backbone. All oligonucleotide sequences used in this study are listed in Table S1. To construct a single-guide RNA (sgRNA) of *MHT1, SAM4, MMP1, URA3, ACS2*, and *MLS1* genes, the amplification of each gene fragment was carried out using Q5 DNA polymerase. NEB restriction enzymes (BclI and SwaI) (New England Biolab, MA, USA) were added and incubated at 37 °C for 2 hr. Then, 3 hr of ligation was performed for using a DNA Ligation Kit (Takara, Kusatsu, Japan).

**Table 1.** Strains and plasmids used in this study

**Strains and plasmids**	**Description**	**Reference**
**Strains**		
BY4742	*MAT⍺ his3*△ *leu2*△ *lys2*△ *ura3*△	Lab stock
CEN.PK	*MATa, his3*△ *leu2*△ *trp1*△ *ura3*△	Lab stock
K6	*S. cerevisiae* Sake Kyokai No. 6, *MATa/⍺*	(Choi et al., 2009)
K6-1	K6 derivative, Leucine auxotroph	(Choi et al., 2009)
BYM	BY4742 harboring p425-*MMT*	This study
CENM	CEN.PK harboring p425-*MMT*	This study
K6M-1	K6-1 harboring p425-*MMT*	This study
K6U-1	K6-1 derivative, *URA3*:: P*_GPD_*_*MMT*	This study
K6U1-1	K6U-1 *MHT1*△, *SAM4*△	This study
K6U2-1	K6U-1 *MMP1*△	This study
K6U3-1	K6U-1 *MHT1*△, *SAM4*△, *MMP1*△	This study
K6U3-1p	K6U3-1 P*_ACS2_*:: P*_PGK1_*	This study
K6U4-1p	K6U3-1p *MLS1*△	This study
**Plasmids**		
p425 GPD	Plasmid with 2µ origin, GPD promoter, CYC1 terminator and *LEU2* selectable marker	Lab stock
p425-MMT	p425GPD harboring *MMT*	This study
pML104	Plasmid with 2µ origin, GAP promoter, CYC1 terminator and *URA3* selectable marker	Lab stock
pML104-Leu	pML104 derivative, *LEU2* selectable marker	This study
pML-*MMT*	pML104-Leu derivative, P_SNR52__tRNA(Gly)_target(*MMT)_*gRNA scaffold	This study
pML-*MHT1*	pML104-Leu derivative, P_SNR52__tRNA(Gly)_target(*MHT1)_*gRNA scaffold	This study
pML-*SAM4*	pML104-Leu derivative, P_SNR52__tRNA(Gly)_target(*SAM4)_*gRNA scaffold	This study
pML-*MMP1*	pML104-Leu derivative, P_SNR52__tRNA(Gly)_target(*MMP1)_*gRNA scaffold	This study
pML-*ACS2*	pML104-Leu derivative, P_SNR52__tRNA(Gly)_target(*ACS2)_*gRNA scaffold	This study
pML-*MLS1*	pML104-Leu derivative, P_SNR52__tRNA(Gly)_target(*MLS1)_*gRNA scaffold	This study

### Growth Medium and Culture Conditions

*Escherichia coli* DH5α was cultured for gene cloning purposes using Luria-Bertani (LB) broth. The cultivation temperature was maintained at 37 °C. For selection, 50 µg/L carbenicillin was added. The initial culture of *S. cerevisiae* K6-1 was performed in 5 mL of yeast extract peptone dextrose medium, comprising 10 g/L of yeast extract, 20 g/L of peptone, and 10 g/L of D-glucose. Strains carrying p425-MMT plasmid were cultured in synthetic defined (SD) medium supplemented with specific amino acids and yeast extract. The SD medium was composed of magnesium sulfate heptahydrate (0.4 g/L), potassium dihydrogen phosphate (4 g/L), dipotassium phosphate (2 g/L), yeast nitrogen base without amino acids (6.7 g/L), and D-glucose (10 g/L). The selected amino acid mixture included L-histidine, L-uracil, and L-tryptophan at a concentration of 10 mg/L. Additionally, 3 g/L of yeast extract was added to the medium. Then, the seed strains were used for inoculation of 5 mL SD medium containing 0.5% (w/v) of glycine and 0.5 g/L of methionine and cultured for 1.5 days. After pre-culturing in the SD medium, 50 mL of fresh SD medium was re-inoculated for the main culture. The entire culture procedure was performed aerobically at 30 ˚C in a shaking incubator at 250 rpm.

Fed-batch fermentation of the SMM-overproducing strain, K6U4-1p, was conducted in 3-L bioreactors, where the working volume of SD medium was set at 1 L, and was continued for 3 days. The medium for fed-batch fermentation is composed of 3% (w/v) of yeast extract, 0.4 g/L of MgSO_4_*7H_2_O, 4 g/L of KH_2_PO_4_, 2 g/L of K_2_HPO_4_, 10 g/L of peptone, 5 g/L of methionine, 5% (w/v) of glycine and 10 g/L of D-glucose. The fermentation process commenced with an initial OD_600_ value of 0.125. During the fed-batch fermentation process, the temperature was maintained at 30 ˚C, with an aeration rate of 1 L/min and an agitation speed of 300 rpm. The aeration rate and agitation speed were increased up to 5 L/min, 500 rpm to maintain the dissolved oxygen over 30%. A solution of 60% (w/v) of D-glucose was used to maintain glucose concentration above 3 g/L.

### Metabolite Assays and RT-PCR

The biomass determination in the cell broth was carried out by measuring the optical density at 600 nm using a DU730 spectrometer (Beckman Coulter, Brea, CA, USA). To analyze the extracellular metabolites, the culture supernatant was obtained by centrifugation. A high-performance liquid chromatography (HPLC) system equipped with a Waters 2414 refractive index detector (Milford, MA, USA) with SH1011 column (Shodex, Tokyo, Japan), was used for the analysis. The detector and oven temperature was set to 45 and 75 °C, respectively, for the quantification of sugars, organic acids, and alcohols. The mobile phase consisted of a 10 mM sulfuric acid solution with a liquid flow rate of 0.6 mL/min.

For the determination of SMM and methionine titers, an Agilent 1260 HPLC system, HPLC UV detector system (Agilent Technology, Santa Clara, CA, USA), was utilized. An amino acid analysis column (Agilent Technology) at an oven temperature of 40 °C was employed, and the HPLC UV system was used for analysis. The mobile phases A and B were used for a gradient flow method, as outlined in Table S2. Mobile phase A consisted of 20 mM Na_2_HPO_4_ with 0.11% (v/v) phosphoric acid, while mobile phase B comprised 45% (v/v) acetonitrile, 45% (v/v) methanol, and 10% (v/v) water. Prior to analysis, the samples were derivatized using OPA reagent (Sigma-Aldrich, St. Louis, MO, USA), and the UV detectors measured the samples at 338 nm.

Similarly, for the determination of SAM titer, an Agilent 1260 HPLC system, HPLC UV detector system was utilized with Eclipse Plus C18 column (Agilent Technology) at oven temperature of 40 °C. The mobile phase A, consisting of 97% 0.5 M ammonium formate with 3% (v/v) methanol, was adjusted for a constant flow rate. The samples were analyzed at 254 nm using an automated injection program with a UV detector.

To compare the expression levels of the ACS2 gene, real-time PCR analysis was conducted. Strains K6U3-1 and K6U3-1p were cultured in SD medium supplemented with yeast extract for 48 hr. Following the harvest of each strain through centrifugation at 3 500 rpm for 5 min, the purification of total RNA was carried out using the RNeasy Purification Kit (Qiagen, Hilden, Germany) in accordance to the manufacturer's instructions. Subsequently, cDNA synthesis was performed using the iScriptTM cDNA synthesis kit (Bio-Rad, Hercules, CA, USA) with 500 ng of total RNA. The resulting cDNA was then utilized as a template for real-time PCR analysis. The PCR was performed using a MiniOpticon (Bio-Rad).

## Results and Discussion

### Identification of Methionine S-methyltransferase and Suitable Yeast Strain

To produce SMM in microorganisms, methionine S-methyltransferase, which is responsible for transferring a methyl group from SAM to methionine, thereby producing SMM and S-adenosylhomocysteine (SAH), is needed. *MMT* genes encoding methionine S-methyltransferases have been identified in several plants, such as mouse ear cress, maize, sunflower, and barley. We synthesized three representative *MMT* genes derived from *A. thaliana, Wedelia bifolora*, and *Hordeum vulgare*, codon-optimized, and expressed them in *E. coli* DH5α. The cultivation of *E. coli* strains was carried out in M9 medium supplemented with 0.5 g/L of methionine for 24 hr. The titer of SMM with *E. coli* containing *A. thaliana*-derived MMT was more than twofold higher than that of the other strains (Fig. S1). Based on these observations, *the MMT* gene from *A. thaliana* was selected for subsequent experiments.

**Fig. 2.**
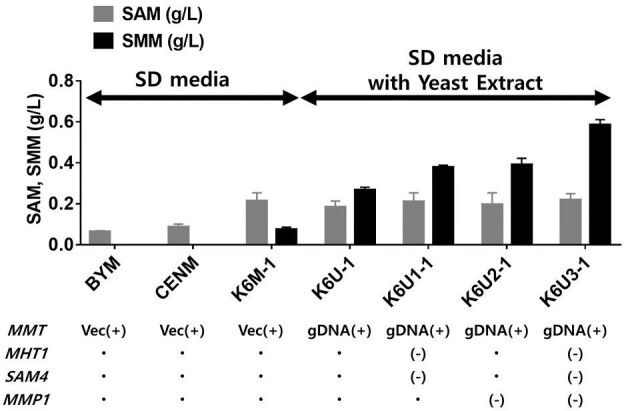
SMM and SAM titer of *MMT* gene introduced by vector for SMM production in BY4742, CEN.PK and K6-1 strains, *MMT* gene integrated in K6-1 strain and various strains that have undergone engineering to increase SMM titer when cultured in a 50 mL flask for 48 hr. Vec(+) indicates overexpressed by vector. gDNA(+) indicates the gene integrated into gDNA for overexpression. (-) indicates the deletion of genes related to SMM production. Error bars represent standard deviations of triplicated experiments.

To using GRAS a generally regarded as safe (GRAS) host, we attempted to produce SMM using the *MMT* gene from *A. thaliana* in yeast. To select an appropriate host, three different yeast strains–BY4742, CEN-PK, and K6-1–were used to produce SMM. The strains carrying p425-MMT, named as BYM, CENM, and K6M-1, were grown in SD medium supplemented with 0.5 g of methionine for 48 hr. As shown in Fig. 2, K6M-1 strain produced considerable amounts of SMM (0.07 g/L), whereas the other two strains did not produce SMM. This was probably due to the ability of the K6-1 strain to produce high levels of SAM, as confirmed in a previous study (Choi et al., 2009).

When heterologous genes are expressed in yeast using plasmids, a defined medium is often used to select the strains with auxotrophic markers. This can cause problems, such as a slow growth rate of the cells and/or low production of target chemicals. To address these issues, the *MMT* gene from *A. thaliana* was integrated into the genome of K6-1 strain, specifically at the *URA3* locus. The resulting strain was named as K6U-1. Through this integration, a specific selection medium is not needed. Additionally, the *URA* auxotrophic phenotype of K6U-1 allows for further genetic engineering. When K6U-1 was cultured in SD medium with yeast extract to alleviate the low specific growth rate (Table S3), a significant increase in SMM production (0.26 g/L) was observed compared to the strain, K6M-1 (Fig. 2). This was more than a threefold increase in SMM production by the integration of genes and by using a complex medium.

### Knock-out of Genes Involved in SMM Degradation Pathway Enhances SMM Titer

Several studies have investigated the SMM degradation pathway in yeast (Rouillon et al., 1999; Thomas et al., 2000; Eder et al., 2022). These studies have successfully identified the genes involved in the uptake of sulfur complexes and those responsible for decomposing and utilizing SMM within the cell. The identification of these genes revealed the biochemical pathways that govern the utilization of sulfur compounds in yeast. Specifically, these studies have shown that yeasts possess specialized transport and degradation pathways for various sulfur compounds, including SMM. Once inside the cell, SMM is degraded by a series of enzymes that produce several compounds that can be used for cell growth and survival.

Two genes in the yeast genome, *MHT1* and *SAM4* that encode homocysteine S-methyltransferases, have been identified in the SMM degradation pathway. Another gene, *MMP1*, which encodes S-methyl-methionine permease, is involved in SMM uptake (Fig. 1). To alleviate SMM degradation, *MHT1* and *SAM4* were knocked out in the K6U-1 strain to produce K6U1-1. In addition, *MMP1* was knocked out to generate K6U2-1 cells. Deletion of Homocysteine S-methyltransferase and S-methyl-methionine permease improved SMM production (Fig. 2). Therefore, the strain with all three genes deleted, K6U3-1, was generated and produced 0.58 g/L of SMM (Fig. 2). We confirmed that knocking out genes related to SMM degradation in yeast effectively enhanced SMM production in yeast.

### Optimizing Precursor Biosynthesis for Improved SMM Production

**Fig. 3.**
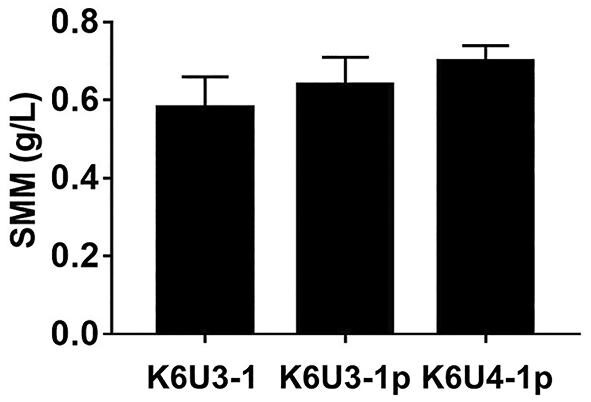
The native *ACS2* promoter was replaced to *PGK1* promoter to up-regulate *ACS2* gene for enhanced acetyl-CoA level. The gene *MLS1* was deleted for enhanced acetyl-CoA level. The following graph illustrates the comparison of SMM production among the strains expected to have increased acetyl-CoA levels. Error bars represent standard deviations of triplicated experiments.

Because SAM is a precursor of SMM, its accumulation can be a key factor in achieving high yields of SMM while minimizing its impact on other SAM-dependent biological processes. Several engineering studies have been carried out to augment the acetyl-CoA pool, which is related to methionine metabolism and to increase the production of SAM (Chen et al., 2016; Kanai et al., 2017). To enhance acetyl-CoA accumulation, the expression of *ACS2* gene encoding acetyl-coenzyme A synthetase was modulated by replacing the native promoter of the *ACS2* gene with the *PGK1* promoter, a strong promoter (Fig. S2). RT-PCR analysis revealed an approximately 1.3fold increase in the expression of *ACS2* (Fig. S2). However, the resulting strain, K6U3-1p, did not exhibit a meaningful increase in SMM production (Fig. 3). We aimed to further increase the pool of acetyl-CoA by blocking its entry into the glyoxylate cycle with deletion of the *MLS1* gene encoding malate synthase in the K6U3-1p strain. According to the previous study (Chen et al., 2016), it was expected that the up-regulation of *ACS2* gene expression and deletion of *MLS1* gene together would lead to a relatively higher intracellular level of acetyl-CoA compared to the wild-type strain, K6-1. The anticipated elevation in acetyl-CoA levels is expected to enhance the efficient accumulation of SAM, subsequently resulting in an increased production of SMM. The resulting strain, K6U4-1p, produced 0.71 g/L of SMM, which is significant increase from that of K6U3-1 (Fig. 3), with slight decrease of the specific growth rate (Table S3). Increasing the intracellular acetyl-CoA pool showed a positive effect in SMM production.

### Overproduction of SMM through Fed-Batch Fermentation

The resulting strain, K6U4-1p, which showed the highest SMM titer, was selected for fermentation to maximize SMM production. For the fed-batch fermentation, a minimal medium, SD media, supplemented with 3% (w/v) yeast extract was utilized. In order to enhance the intracellular production of SMM, the folate cycle, which plays a vital role in SAM biosynthesis, was effectively stimulated by supplementing of 0.5% (w/v) glycine. Methionine (5 g/L) was added to promote SMM production. A 1 L medium was used in a 3 L-jar for fed-batch fermentation. d-Glucose, with an initial concentration of 10 g/L, was monitored every hour to maintain at least 3 g/L of glucose by feeding.

**Fig. 4.**
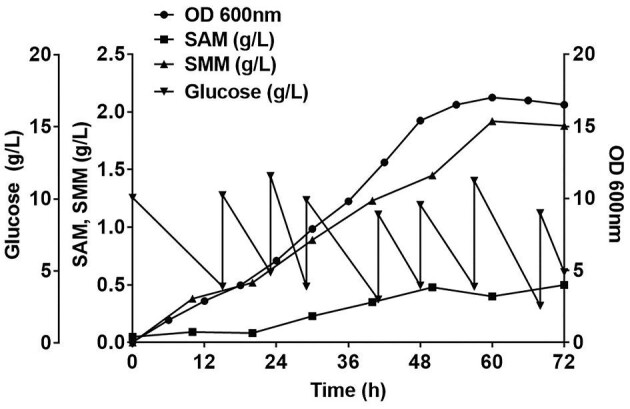
Results of 72 hr fed-batch fermentation with 1 L working volume. D-glucose (10 g/L), 3% (w/v) of yeast extract, 0.5% (w/v) of glycine and 5 g/L of methionine were used for initial media. The SMM overproducing strain, K6U4-1p, was used for SMM synthesis. Black circle, cell growth; Black square, SAM (g/L); Black triangle, SMM (g/L); and Black reverse triangle, Glucose (g/L)

The maximum optical density (OD_600_) of the fermentation reached 16.8 at 72 h. The highest SMM titer in fermentation was 1.92 g/L, which was achieved through periodic feeding of D-glucose during fed-batch fermentation (Fig. 4). This is significant, considering that this is the first attempt at SMM production by microorganisms.

## Conclusion

We attempted the heterologous production of SMM in a GRAS microorganism by introducing a key enzyme, *MMT*, from *A. thaliana*. The *MMT* gene was synthesized by codon optimization in *S. cerevisiae* and integrated into the *URA3* locus using CRISPR-Cas9. *S. cerevisiae* K6-1 strain, with enhanced intracellular SAM production, successfully produced a significant amount of SMM. To enhance SMM production, genes involved in SMM recycling (*MMP1, MHT1*, and *SAM4*) were knocked out in the host strain. Furthermore, the expression of *ACS2* was enhanced by substituting its promoter, and the removal of *MLS1* was carried out to augment the intracellular pool of acetyl-CoA, the precursor of SAM. The final strain, K6U4-1p, was used in fed-batch fermentation, which resulted in an SMM titer of approximately 2 g/L. This is the first report of SMM production from a microorganism and demonstrates the potential of genetic engineering to enhance SMM production in *S. cerevisiae*.

## Supplementary Material

kuad026_Supplemental_File

## Supplementary Material

The following information is included in the Supporting Information: All primers and sequences used in this study and flow rate for each mobile phase as a function of time in HPLC for measuring SMM production. Specific growth rates and titers of the engineered strains in different media. The results of the SMM titers using various plant-derived *MMT* genes expressed in *E. coli* are presented in the data. The data included in this study comprised the growth curves for each strain. Additionally, the results of RT-PCR analysis after replacing the native *ACS2* promoter with the well-known strong *PGK1* promoter are provided.
