# Supplementary material for: Production of S-methyl-methionine using engineered Saccharomyces cerevisiae sake K6
Source: J Ind Microbiol Biotechnol. 2023 Aug 31;50(1):kuad026. doi: 10.1093/jimb/kuad026 (PMC10495038; doi:10.1093/jimb/kuad026)
Supplement: kuad026_Supplemental_File [file kuad026_supplemental_file.docx]

**Supporting Information**

Production of S-methyl-methionine using engineered *Saccharomyces cerevisiae* sake K6

Jun-Min Lee^1,†^_,_ Min-Ho Park^1,†^, Bu-Soo Park^1,†^_,_ Min-Kyu Oh^1,^*

^1^ Department of Chemical & Biological Engineering, Korea University, Seoul 136-763, Korea

† These authors contributed equally to this work.

^*^ Corresponding author. **Min-Kyu Oh** ([mkoh@korea.ac.kr](mailto:mkoh@korea.ac.kr))

**Author information**

**Corresponding Author**

**Min-Kyu Oh** - Department of Chemical and Biological Engineering, Korea University, Anam-Ro 145 Sungbuk-Gu, 02841 Seoul, Korea; orcid.org/0000-0001-6406-3930; Tel.: +82-2-3290-3308; E-mail: [mkoh@korea.ac.kr](mailto:mkoh@korea.ac.kr); Fax: +82- 2-926-6102.

**Authors**

**Jun-Min Lee**, Department of Chemical and Biological Engineering, Korea University, Anam-Ro 145 Sungbuk-Gu, 02841 Seoul, Korea

**Min-Ho Park**, Department of Chemical and Biological Engineering, Korea University, Anam-Ro 145 Sungbuk-Gu, 02841 Seoul, Korea

**Bu-Soo Park**, Department of Chemical and Biological Engineering, Korea University, Anam-Ro 145 Sungbuk-Gu, 02841 Seoul, Korea

**Table S1**. Primers and sequences used in this study.

|  | **Target sequence** | **Primer F** | | **Primer R** |
| --- | --- | --- | --- | --- |
| **MHT1** | ATGTTGCACGAGCATCTACC | GAT CCT CTA ATG CGC AAG TGG TTT AGT GGT AAA ATC CAA CGT TGC CAT CGT TGG GCC CCC GGT TCG ATT CC | | CTA GCT CTA AAA CGG TAG ATG CTC GTG CAA CAT TGC GCA AGC CCG GAA TCG AAC CGG GGG CCC AAC GAT GGC AAC |
| **MMP1** | TCGACATTAGGGATATACGG | GAT CCT CTA ATG CGC AAG TGG TTT AGT GGT AAA ATC CAA CGT TGC CAT CGT TGG GCC CCC GGT TCG ATT CC | | CTA GCT CTA AAA CCC GTA TAT CCC TAA TGT CGA TGC GCA AGC CCG GAA TCG AAC CGG GGG CCC AAC GAT GGC AAC |
| **SAM4** | GACAAATATGAGCACCCCAT | AAC TTC TCC GCA GTG AAA GAT AAA TGA TCC TCT AAT GCG CAA GTG GTT TAG TGG TAA AAT CCA ACG TTG CCA TCG TTG GGC CCC CGG TTC GAT TCC GGG CTT | | CTA GCC TTA TTT TAA CTT GCT ATT TCT AGC TCT AAA ACC ACG GGA TTT GCA ACT TTG ATG CGC AAG CCC GGA ATC GAA CCG GGG GCC CAA CGA TGG CAA CGT |
| **URA3** | TTGATTATGACACCCGGTGT | AAC TTC TCC GCA GTG AAA GAT AAA TGA TCC TCT AAT GCG CAA GTG GTT TAG TGG TAA AAT CCA ACG TTG CCA TCG TTG GGC CCC CGG TTC GAT TCC GGG CTT | | CTA GCC TTA TTT TAA CTT GCT ATT TCT AGC TCT AAA ACA CAC CGG GTG TCA TAA TCA ATG CGC AAG CCC GGA ATC GAA CCG GGG GCC CAA CGA TGG CAA CGT |
| **ACS2** | TGTATAACAATCACTAACCG | AAC TTC TCC GCA GTG AAA GAT AAA TGA TCC TCT AAT GCG CAA GTG GTT TAG TGG TAA AAT CCA ACG TTG CCA TCG TTG GGC CCC CGG TTC GAT TCC GGG CTT | | CTA GCC TTA TTT TAA CTT GCT ATT TCT AGC TCT AAA ACC GGT TAG TGA TTG TTA TAC ATG CGC AAG CCC GGA ATC GAA CCG GGG GCC CAA CGA TGG CAA CGT |
| **MLS1** | ATAGGTCAACGGAAATCACAGGG | AAC TTC TCC GCA GTG AAA GAT AAA TGA TCC TCT AAT GCG CAA GTG GTT TAG TGG TAA AAT CCA ACG TTG CCA TCG TTG GGC CCC CGG TTC GAT TCC GGG CTT | | CTA GCC TTA TTT TAA CTT GCT ATT TCT AGC TCT AAA ACC GGT AGT AAT CTC ACC ATT TTG CGC AAG CCC GGA ATC GAA CCG GGG GCC CAA CGA TGG CAA CGT TGG ATT TT |
|  | | | **Sequence** | |
| **tRNA_Gly** | | | CTCTAATGCGCAAGTGGTTTAGTGGTAAAATCCAACGTTGCCATCGTTGGGCCCCCGGTTCGATTCCGGGCTTGCGCA | |
| **gRNA scaffold** | | | GTTTTAGAGCTAGAAATAGCAAGTTAAAATAAGGCTAGTCCGTTATCAACTTGAAAAAGTGGCACCGAGTCGGTG | |

**Table S2**. Gradient flow and derivatization using OPA reagent for HPLC analysis

| Time (min) | Mobile phase A (%) | Mobile phase B (%) |
| --- | --- | --- |
| 0 | 98 | 2 |
| 1.9 | 98 | 2 |
| 18.1 | 43 | 57 |
| 18.6 | 20 | 80 |
| 22.3 | 20 | 80 |
| 23.2 | 98 | 2 |
| 26.0 | 98 | 2 |
| Order | Procedure |  |
| 1 | Add 20 μL borate buffer to sample | |
| 2 | 30s wait | |
| 3 | Add 5 μL of OPA reagent to sample | |
| 4 | 30s wait | |
| 5 | Mix 10 μL volume for 5 times | |
| 6 | Inject 20 μL | |

**Table S3.** The following table presents the specific growth rates and SMM titers with standard deviations (in parenthesis) of triplicated experiments for the engineered strains under two conditions: using SD media alone and supplementing SD media with yeast extract.

| Media | Strain | Specific Growth rate  (Standard deviation) (/h) | Product (SMM) titer  (Standard deviation) (g/L) |
| --- | --- | --- | --- |
| SD media | BYM | 0.092 (0.0062) | - |
|  | CENM | 0.117 (0.0085) | - |
|  | K6M-1 | 0.129 (0.0106) | 0.07 (0.0117) |
| SD media with Yeast extract | K6U-1 | 0.182 (0.0061) | 0.26 (0.0138) |
|  | K6U1-1 | 0.176 (0.0061) | 0.39 (0.0095) |
|  | K6U2-1 | 0.191 (0.0055) | 0.41 (0.0309) |
|  | K6U3-1 | 0.198 (0.0135) | 0.58 (0.0544) |
|  | K6U3-1p | 0.181 (0.0050) | 0.63 (0.0506) |
|  | K6U4-1p | 0.175 (0.0036) | 0.71 (0.0271) |


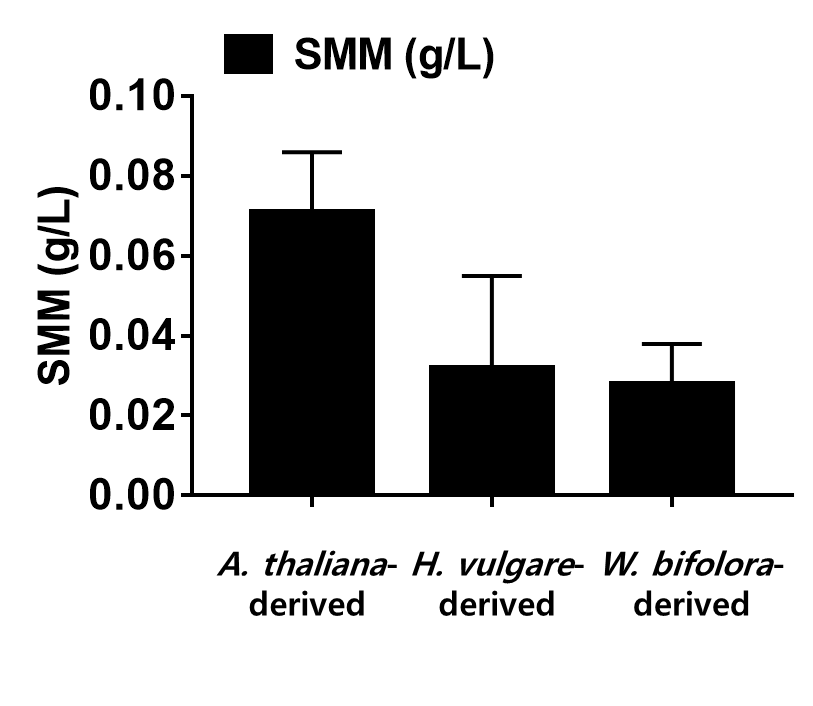


**Fig S1.** To determine which plant-derived *MMT* gene was introduced, we first tested the expression of three different *MMT* genes (*Arabidopsis thaliana, Hordeum vulgare, Wedelia bifolora*) in *E. coli* and measured their SMM titers. The strain expressing the *A. thaliana*-derived *MMT* gene had the highest SMM titer. Error bars represent standard deviations of triplicated experiments.


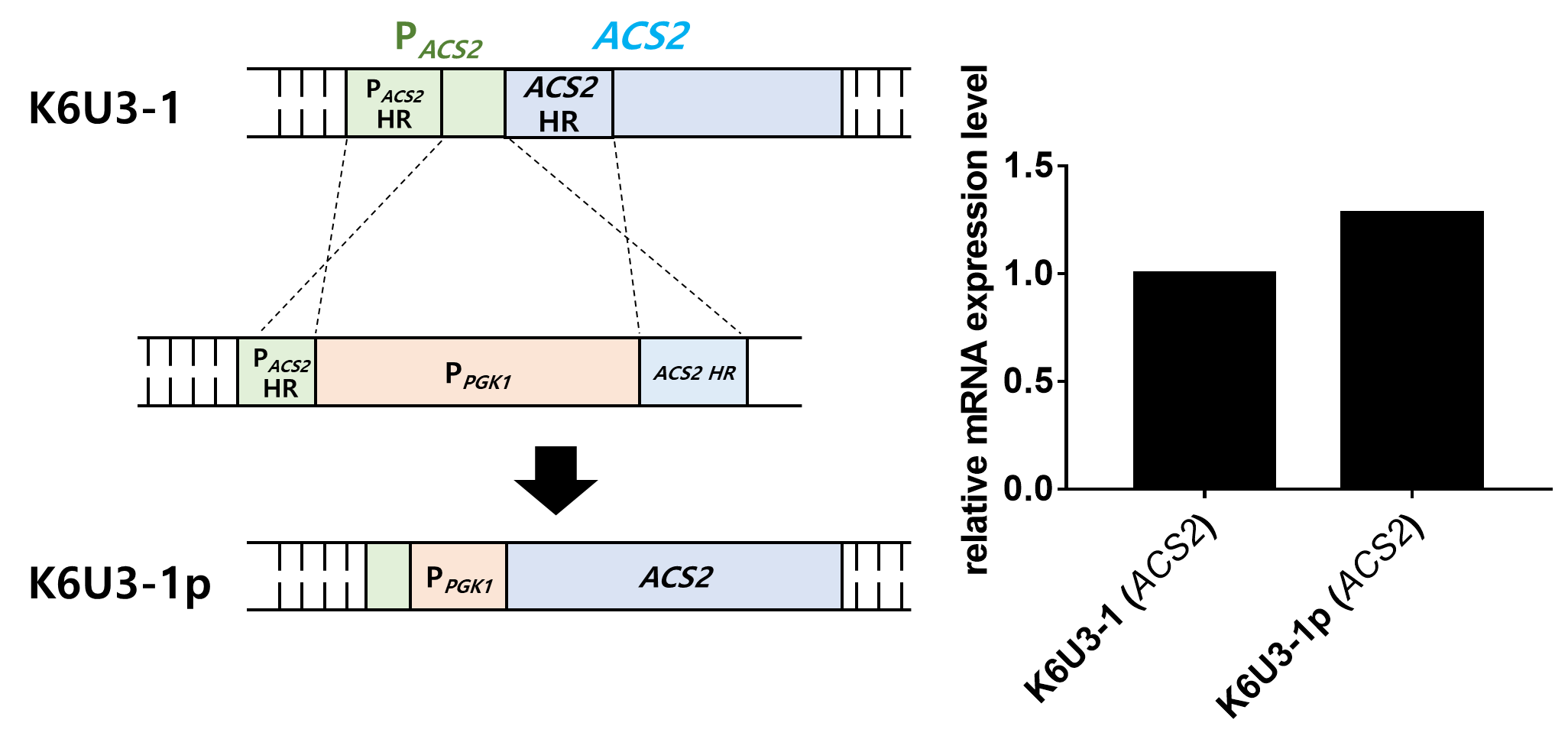


**Fig S2.** The native *ACS2* promoter was replaced with the well-known *PGK1* promoter. RT-PCR analysis was conducted to measure the relative mRNA expression levels of the strain carrying the enhanced *ACS2* promoter and the strain without it. The results showed an approximately 1.3-fold increase in mRNA expression compared to the K6U3-1 strain.
